# Alternative Materials from Agro-Industry for Wood Panel Manufacturing—A Review

**DOI:** 10.3390/ma15134542

**Published:** 2022-06-28

**Authors:** Nicolas Neitzel, Reza Hosseinpourpia, Thomas Walther, Stergios Adamopoulos

**Affiliations:** 1 Department of Forestry and Wood Technology, Linnaeus University, Georg Lückligs Plats 1, 35195 Växjö, Sweden; nicolas.neitzel@lnu.se (N.N.); reza.hosseinpourpia@lnu.se (R.H.); 2 IKEA Industry AB, Skrivaregatan 5, 21532 Malmö, Sweden; thomas.walther@inter.ikea.com; 3 Department of Forest Biomaterials and Technology, Swedish University of Agricultural Sciences, Vallvägen 9C, 75007 Uppsala, Sweden

**Keywords:** agricultural residues, wood panels, particleboard, straw, stalks, sustainability

## Abstract

The growing demand for wood-based panels for buildings and furniture and the increasing worldwide concern for reducing the pressure on forest resources require alternatives to wood raw materials. The agricultural industry not only can provide raw materials from non-wood plants but also numerous residues and side streams. This review supplies an overview of the availability, chemical composition, and fiber characteristics of non-wood lignocellulosic materials and agricultural residues, i.e., grow care residues, harvest residues, and process residues, and their relevance for use in wood panel manufacturing. During the crop harvest, there are millions of tons of residues in the form of stalks, among other things. Usually, these are only available seasonally without using storage capacity. Process residues, on the other hand, can be taken from ongoing production and processed further. Fiber characteristics and chemical composition affect the panel properties. Alternatives to wood with long fibers and high cellulose content offer sufficient mechanical strength in different panel types. In general, the addition of wood substitutes up to approximately 30% provides panels with the required strength properties. However, other parameters must be considered, such as pressing temperature, adhesive type, press levels, and pretreatments of the raw material. The search for new raw materials for wood panels should focus on availability throughout the year, the corresponding chemical requirements and market competition. Panel type and production process can be adapted to different raw materials to fit niche products.

## 1. Introduction

Sustainable management and the use of raw materials have become increasingly important during the last decades. The global interest and online search for sustainable products have grown by over 71% since 2016 [1]. The world population is projected to reach 8.5 billion in 2030 and increase further. This goes hand in hand with an increasing demand for living space and thus for building and furniture materials [2]. Given the growing global demand for sustainable products, the pressure on the forestry sector as a main source of renewable raw materials is more significant than ever [3].

Likewise, biodiversity, the quantity and quality of forests, and their protection are integral parts of current global and regional policies, for example, in the European Union Biodiversity Strategy for 2030 or the United Nations Sustainable Development Goals (SDGs) [4]. This, together with the increasing timber prices, as well as delivery difficulties due to affected logistics, urge the wood panel industry to look for alternative raw material sources. 

Non-wood lignocellulosic materials (NWLM) and agriculture residues (AR) can be promising alternative raw materials for the wood industry since they originate from renewable sources and are widely available [5]. ARs are often burned for energy production [6] or used as animal feed [7] and as a natural fertilizer left in the fields. Some ARs are also partially burned in the fields since removal or mechanical incorporation into the soil is economically inviable [8]. However, since combustion releases greenhouse gases and causes high levels of air pollution, the field burning of agricultural waste or residues is not permitted in most of the European Union’s member states, among other places [9].

Valorizing the ARs into value-added products, such as in wood panel manufacturing (mainly particleboards and fiberboards), increases their value and brings ecological benefits. Simultaneously, it relieves the pressure on virgin forest raw materials. Although there are various studies on the utilization of NWLMs and ARs for wood-based panels [10,11,12,13] and reviews that collected and summarized the findings [5,14,15,16], there is still a lack of information about the critical requirements of these materials to serve as a partial or entire replacement of wood. 

Some material properties, such as the anisotropic and hygroscopic characteristics of NWLM and ARs are similar to that of wood, while their density is generally lower [14]. This brings an advantage for producing low-density composites. The wood particles or fibers cannot be replaced with alternative materials in a one-to-one ratio due to their low mechanical strength and high proportion of fines [17]. Therefore, the application of alternative materials in panel manufacturing is only feasible up to a certain amount in the presence of wood. Otherwise, higher amounts of adhesives are required to meet the required mechanical strength level of the panels. Although massive amounts of NWLMs and ARs exist worldwide, the majority of those are only seasonal and not evenly available throughout the year [18].

**Figure 1:**
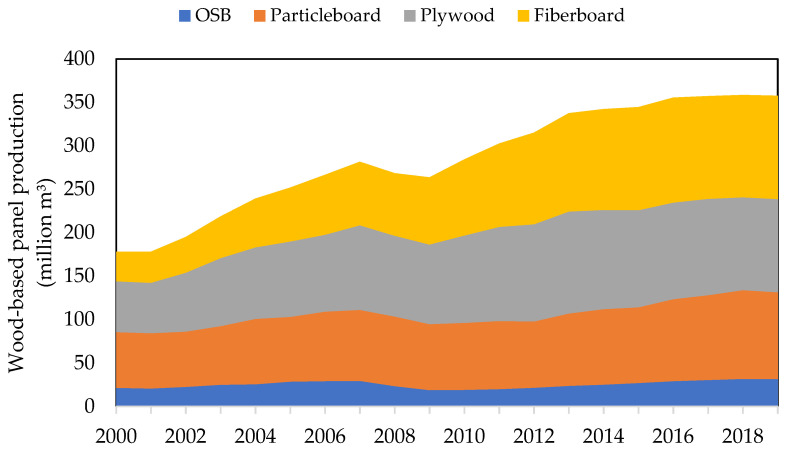
Production volume of wood-based panels from 2000–2019 worldwide [19].

In the past 20 years, the production volume of wood-based panels has almost doubled from approximately 180 million m^3^ in 2000 to over 361 million m^3^ in 2020 [19]. The share of oriented strand boards (OSB) and plywood production increased only slightly in the period. On the other hand, the production volume of particleboard and especially fiberboard panels has increased significantly by 32% (Figure 1). Considering an average density of 750 kgm^−3^ for each fiberboard panel, about 184 million tons of raw lignocellulosic materials are required to meet this production volume without considering the required adhesives and production losses. 

According to the Food and Agricultural Organization (FAO), about 850 million tons of residues (wheat stalks and husks) were incurred in European agricultural operations in 2018 [19]. The large number of available NWLMs and ARs may cover some part of the demands for raw materials for the wood panel industry. Nevertheless, the potential expected volume of materials depends on the geographical region. For instance, Europe has a forest area of over 10.17 million km^2^ and a cropland area of about 2.89 million km^2^, while these areas in Asia are 6.24 and 5.90 million km^2^, respectively (Table 1).

This article presents a review of the research performed on using alternative NWLMs and ARs from the agricultural industry for wood panel manufacturing. It encompasses information on raw material categories and availability, their fiber and chemical characteristics, and utilization in panel manufacturing with the partial or entire replacement of wood. The performance of manufactured panels at various production parameters is included as a common requirement in the initial development stages when using new materials. The pretreatment and processing of these raw materials are also discussed. The opportunities and challenges of using such alternative materials are described, and promising materials for further investigation are proposed. 

## 2. Categories of Different Alternative Materials

**Figure 2:**
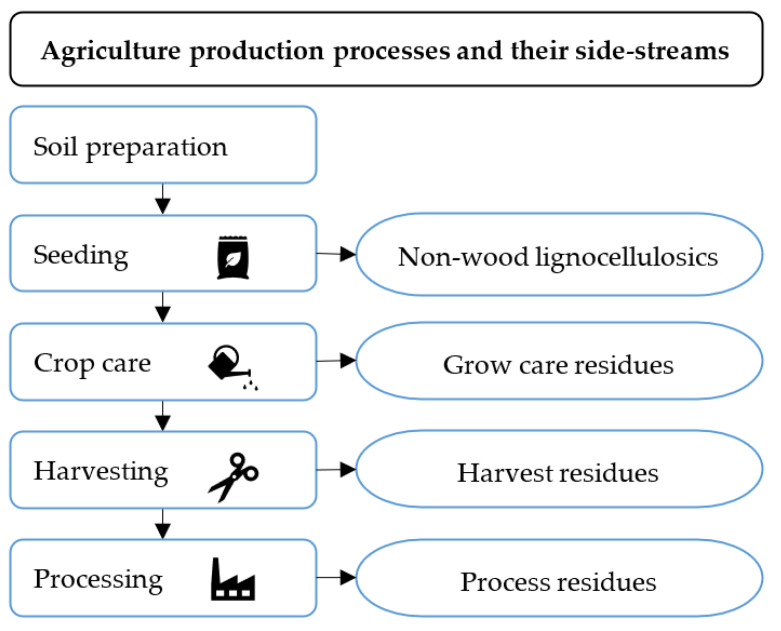
Different agriculture production processes and their related available side-streams.

A variety of lignocellulosic materials from various sources and agricultural production processes have received attention as alternative raw materials for wood-based panel manufacturing and mainly include by-products, side streams, and residues. These materials can be further categorized as non-wood lignocellulosic materials and three types of agricultural residues, i.e., grow care residues, harvest residues, and process residues (Figure 2).

**Non-wood lignocellulosic materials (NWLM)** are derived from crops primarily cultivated for use in the food and textile industry. They can be found around the world but to a varying degree. About 1.1 million tons of fibers are produced annually from growing flax, of which 97.1% are from Europe [19]. Due to the legislative approval and increasing interest in cannabidiol, hemp cultivation in the United States has also increased enormously since 2018 [20]. Only in 2019, about 3.4 million tons of jute and 0.2 million tons of sisal were produced worldwide [19]. 

Flax and hemp are mainly cultivated because of their seeds to produce oil in the food industry [21,22], while their fiber, together with kenaf and sisal, are used in textiles (clothes, mattresses, ropes, etc.) due to their high length, strength, and durability [23,24]. Kenaf fibers are commercially used as an insulating material in constructions. The NWLM category also includes ornamental plants that are grown for decorative purposes, such as rhododendrons or alternatives like bamboo. The fast-growing grass bamboo is used for various applications, such as construction, food, biofuel, pulp, and panel making. This diversity is mainly due to its considerable growing number annually, which is over 0.32 million km^2^ worldwide [25].

**Grow care residues** are the first group of agricultural residues from plant materials and arise during crop maintenance. When fruits grow, the plants are pruned to allow the fruit bodies to reach the ideal growth [26]. There is no economic use proposed for this biomass type rather than burning, i.e., thermal use of apple and olive tree pruning [27,28], or an attempt to produce ethanol from it, i.e., ethanol production from olive tree pruning [29]. There are no accurate numbers for available existing materials from grow care residues as they are not measured in most cases. 

**Harvest residues** or primary residues are mainly stalks, straws, leaves, sticks, and roots. These materials are collected during the harvest of cereals or other crops, and they are mainly used for animal feed, bedding animals, or in pallet form as an energy source [30]. However, most of this material type is left in the field without further application, which can sometimes lead to disposal problems for farmers [31]. The terms stalks, straw, and sticks are named stalks hereafter. A ton of rice, wheat, oat, and rye harvest produces about 1.3–1.6 tons of stalks. These numbers for cotton and sorghum harvesting are about 3.4 and 2.4 tons, respectively [19]. The quantity of harvest residues can be assessed by considering the residue-to-crop ratio through a ton of the produced main product, i.e., wheat grain, of a specific cultivated plant. The average residue-to-crop ratio of available harvest residues in Europe and worldwide is presented in Table 2.

**Process residues,** including agro-industrial residues or secondary residues, are created when the plants are processed from the primary resource. Husks, hulls, peels, coir, bagasse, and skins are produced during the processing of the main product. The terms husk, shell, and hull can be used interchangeably [32] or with different meanings [33]. Since there is no standard terminology, all main crop’s protective surrounding materials are named husks in this work. Husk material is first produced in the field during the harvest and can also be collected during mechanical cleaning in industrial processing. Different products are obtained during the processing of cereal grain, for instance, husks, flour, and bran. 

The bran is a combination of ground husks and flour. Most of these residues are used as animal feed. However, due to their high fiber content, they can also be consumed by humans and are considered to be healthy [34]. As a food source, it is estimated that the consumption alone is about 90 million tons per year [35]. Bagasse is a side-stream of sugarcane stalks, and it is mainly used as an energy source in factories [36]. In addition to being used as fodder, it is also a raw material in the pulp and paper industry [37]. 

However, since the annual quantities are enormous and not everything is used, there is a huge potential for creating added value from bagasse and other ARs than solely used for thermal incineration. Harvesting one ton of soybeans produces around 1.09 tons of husks (Table 2). The quantities for producing a ton of coffee and rice are respectively 1.32 and 0.25 tons [19]. The exact conversion factors can vary widely due to different influencing elements, such as soil conditions, weather, and the harvesting process [38]. In addition, growth phases in northern regions are shorter than in regions near the equator.

### 2.1. Characteristics of Alternative Materials

The performance of composite panels depends greatly on the characteristics of their constituents. The chemical composition and fiber morphology of lignocellulosic materials from the agricultural industry vary considerably with the plant species, age, climate, and soil conditions. The individual species in a plant family can also show different chemical composition and fiber morphology. There are, for example, many different types of bamboo or rice and sunflower varieties. In order not to list each species individually, plant families were grouped and a range of their chemical constituents and fiber morphology is given (Table 3 and Table 4).

#### 2.1.1. Chemical Composition of Alternative Materials

As an organic material, wood is mainly composed of cellulose, hemicelluloses, lignin, extractives, and some minerals [39]. Hardwoods consist of 42–49% cellulose, 24–30% hemicelluloses, 25–30% lignin, 2–9% extractives, and 0.2–0.8% minerals/ ash. In contrast, softwoods contain 42–51% cellulose, 27–40% hemicelluloses, 18–24% lignin, 1–10% extractives, and 0.2–0.8% ash [40]. The chemical composition of different alternative furnish materials is summarized in Table 3. For classic wood-based panels, it has been well described previously how the chemical composition of the raw material influences the properties of the manufactured panels. Cellulose and hemicelluloses are the skeleton and backbone of the wood. Accordingly, a high level of strength is achieved with a high cellulose content [41]. At the same time, hemicelluloses lead to water absorption because of their hydrophilic properties. 

Lignin and extractives tend to be more hydrophobic in nature. It reduces water absorption of the panels and thickness swelling [42]. Extractives can also have various other impacts on the panel properties. Depending on the extractive type and share, they can influence the bonding behavior of common synthetic adhesives, lead to low or higher formaldehyde emissions or even improve the bonding behavior (i.e., tannins) in the panel [43]. The amount of ash also influences the bond quality. Ash components have no wettability, which can cause poor adhesive distribution [44].

**Table 3:** Chemical composition (%) of alternative NWLMs and agricultural residues (black bars) as compared with wood (softwoods and hardwoods combined, and green background bars), adopted from references.

Material	References	Chemical Composition, (%)				
		Cellulose	Hemicelluloses	Lignin	Extractives	Ash
**NWLM**		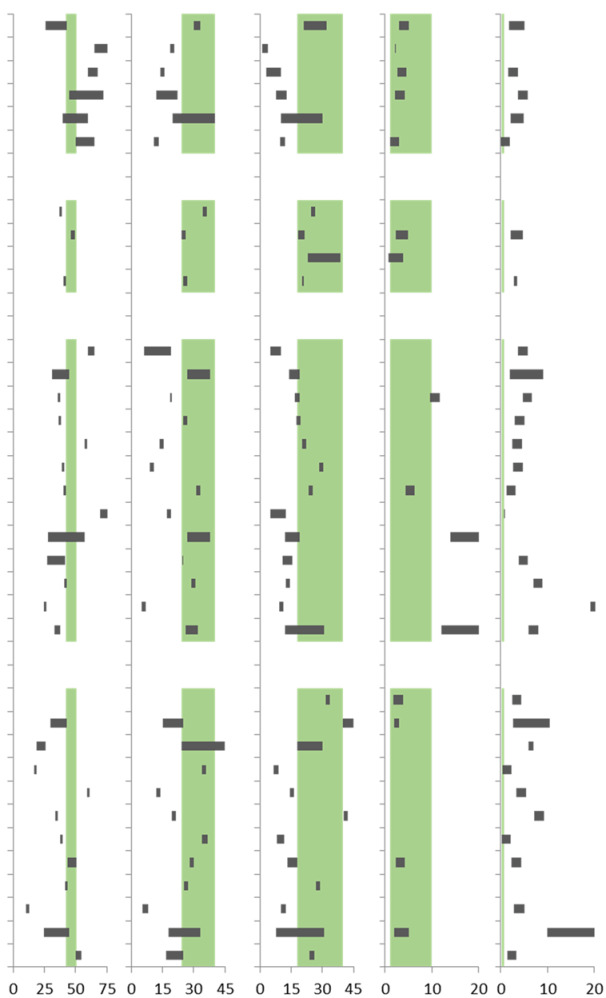				
Bamboo	[45,46,47,48]					
Flax	[45,47,48]					
Hemp	[45,47,48]					
Kenaf	[45,47,48]					
Miscanthus	[49]					
Sisal	[45,48,50]					
	
**Grow care residues**						
Kiwi pruning	[51]					
Orange pruning	[52]					
Pinecone	[53]					
Vine pruning	[54,55]					
	
**Harvest residues**						
Banana wood	[45,46,47,48]					
Barley stalks	[56]					
Canola stalks	[57]					
Corn stalks	[58]					
Cotton stalks	[55]					
Date palm	[59]					
Oil palm	[60]					
Pineapple leaves	[50,56]					
Rice stalks	[45,56]					
Sorghum stalks	[55,56]					
Sunflower stalks	[55]					
Tomato stalks	[58]					
Wheat stalks	[45,56]					
	
**Process residues**						
Almond husks	[61]					
Coconut coir	[45,47,48,56]					
Coffee husks	[33]					
Corn husks	[58]					
Durian peel	[62]					
Hazelnut husks	[63]					
Oat husk	[64]					
Oil palm fruit husks	[59,60]					
Peanut husks	[63]					
Pineapple peel	[65]					
Rice husks	[45,48]					
Sugarcane bagasse	[45,66]					

Although the NWLMs contain similar components as wood, their proportion varies. For instance, flax and hemp have a considerably higher cellulose content than wood, i.e., the respective cellulose contents in flax and hemp are 65–85% and 60–68%, while their lignin content is obviously lower, i.e., flax has 1–4% lignin, and hemp has 3–10% [45,67]. Miscanthus, however, has a similar chemical composition to wood by having 40–60% cellulose, 20–40% hemicelluloses, 10–30% lignin, and 2.2–4.9% ash content [49]. This is also valid for different types of bamboo grasses [45,46,47,48]. 

The grow care residues from pruning and trimmings mostly have similar properties compared to their fruit plants [54,68]. As an example, kiwi pruning composes of 38.3% cellulose, 35.2% hemicelluloses, and 25.5% lignin while vine pruning contain 41.4% cellulose, 26% hemicelluloses, and 20.3% to 21.0% lignin [51,54,55]. 

The cellulose and lignin contents of harvest residues, such as canola, corn, or wheat stalks, are lower than that of wood, i.e., the cellulose content of cereal stalks is approximately between 27% to 38%, and their lignin amount is generally between 12% to 31%. However, their hemicelluloses content is approximately 19% to 38%, which is in the range of wood [45,55,56,57,58]. Stalk materials commonly contain high levels of extractives such as waxes, fats, terpenes, and phenols [39,69]. Their ash content is up to ten times higher than wood [58]. For example, the ash content of wheat stalks is 6–8% [45]. The harvest residues generally have a significant amount of inorganic elements. In some cases, like tomato stalks, the ash content can reach up to 20% of their composition [48,58,64]. Canola stalks with 4.7–6.7% and barley stalks with 2–9% also have a significantly higher ash content than wood [56,57]. 

Among different types of process residues, sugarcane bagasse has a closer amount of hemicellulose, lignin, and ash content to that of wood, while its cellulose content is considerably higher. Pandey et al. [66] and Faruk et al. [45] reported that sugarcane bagasse has 50–55% cellulose, 16.8–25% hemicellulose, 24–26.3% lignin, and 1.4–3.4% ash. The chemical composition of process residues is mainly influenced by annual growth conditions and regions [70]. 

The husks of cereals usually have low cellulose content, i.e., the respective cellulose content in corn, oat, and rice husks are 18%, 38.7%, and 25–45%, and vary with the growth conditions. Nevertheless, the ash content is generally higher than wood, which may cause some limitations for their processing by reducing the service life of machinery, i.e., tool wear, cutting or grinding machines [14,71,72]. A high ash content might be advantageous for specific applications. Beh et al. [73] showed recently that the use of wood ash in a coating of steel beams increases fire resistance.

#### 2.1.2. Fiber Characteristics of Alternative Materials

The morphology of the fibers is essentially relevant for their application in fiber form in relevant wood-based panels (i.e., fiberboards). However, if NWLM or ARs are used in particle form, the particle properties are also influenced by the fiber structure. For the production of fiberboards, long fibers with a higher aspect ratio are preferred [42]. Long fibers provide a larger surface area, allowing the adhesive to spread more evenly. 

At the same time, it allows for more contact surfaces and overlaps between the fibers [74]. Also, long fibers, compared to short ones, tend to arrange themselves horizontally in the mat during panel production rather than vertically. This has a positive effect on the bending behavior of the panels. Fiber diameter and density are closely related to the cell-wall thickness. A thin cell wall allows the fiber to deform more flexible without breaking. 

This, in turn, leads to more contact areas with other fibers within the panel. Thick cell walls, therefore, tend to reduce the bending properties [75]. The fibers from NWLMs are generally longer than wood fibers and therefore have a higher surface area (Table 4). For instance, the respective length of flax and hemp fibers are 10–65 mm [67] and 5–55 mm [76], while the fiber lengths in softwoods and hardwoods are approximately 2.8–7.2 mm [77] and 0.3–2.5 mm [76], respectively. The densities of flax and hemp fibers are approximately 1.4–1.5 gcm^−3^ [48], which is similar to that of wood fibers [78]. With a similar density of 1.45 gcm^−3^ to wood, sisal also has comparable fiber lengths of 0.8–8 mm [67]. In contrast, bamboo fibers with a length of 1.5–4.4 mm and diameter of 7–27 µm have a density of 0.6–1.1 gcm^−3^. The length of bamboo fibers is comparable to miscanthus fibers, with a length of 0.81–1.05 mm [79].

**Table 4:** Fiber characteristics of alternative materials as compared with wood fibers.

Material	Fiber Characteristics			
	Length (mm)	Diameter (µm)	Density (gcm^−3^)	References
**Wood**	0.3–7.2	10–45	1.4–1.5	[48,76,77,78,80]
**NWLM**				
Bamboo	1.5–4.4	7–27	0.6–1.1	[48,76]
Flax	10–65	5–38	1.4	[67]
Hemp	5–55	1–5	1.4–1.5	[48,76,81]
Kenaf	3.55–5.5	12–37	1.4	[48,67,82]
Miscanthus	0.81–1.05	11.8–16.7	-	[79]
Sisal	0.8–8	7–47	1.45	[50,67]
**Grow care residues**				
	n.a.	n.a.	n.a.	
**Harvest residues**				
Banana wood	0.17	13.6	1.35	[50,67]
Canola stalks	1.22	28	-	[83]
Corn stalks	1.22	24.3	-	[84]
Cotton stalks	0.84	23.9	1.45–1.85	[83,85]
Oil palm wood	0.66	29.6–35.3	0.7–1.55	[50,67,86]
Pineapple leaves	-	20–80	1.526	[87,88]
Rice stalks	0.4–3.4	4–16	0.38	[89,90]
Sorghum stalks	1.8	13.8	-	[91]
Sunflower stalks	1.18	21.5	0.154	[84,92]
Tomato stalks	0.83–1.13	13.24–17.26	0.58	[93,94]
Wheat stalks	1.1–1.13	11.9–15.3	-	[95]
Barley stalks	0.7–3.1	7–24	-	[56]
**Process residues**				
Coconut coir	20–150	10–460	1.15	[81]
Coffee husks	0.05–0.8	15	-	[33]
Corn husks	0.5–1.5	10–20	-	[56]
Durian peel	0.84–2.38	170–447	1.15–1.31	[96]
Oil palm fruit husks	0.89–0.99	19.1–25	0.7–1.55	[67]
Rice husks	-	170	1.16	[97,98]
Sugarcane bagasse	1.59	20.96	0.99	[97,99]

There is limited data available on fiber dimensions from grow care residues. Ntalos and Grigoriou [54] reported that the anatomical and chemical components of the grow care residues correspond to those of the main plant. In comparison with wood and NWLMs, the harvest residues have noticeably shorter fibers, as their fiber lengths are mainly ranged from 1.1 mm for wheat stalks [95] to 1.8 mm for sorghum stalks [91], which is about half of wood fibers. The fiber diameter is, on average, approximately 30% smaller than wood [83,84,92]. The fiber morphology of harvest residues could, therefore, have a negative impact on the bending properties. 

As with the chemical composition, the fiber dimensions of process residues are also varied due to growth and processing conditions and some individual settings. However, since these materials are collected after the processing of the crops, their fibers are generally shorter than wood fibers [100]. The length of fibers from different husks, such as coffee husks or oil palm fruit husks, ranges from 0.05 to 0.99 mm [33,56,67]. With 15 µm, 10–20 µm, and 19.1–25 µm, the fiber diameters of coffee, corn, and oil palm fruit husks is roughly half that of wood fibers [33,56,67]. Due to the clearly shorter dimensions, the length-width ratio of process residues differs from wood fibers, as they have a cubic form. For example, coconut coir and rice husks have with their large fiber diameters of 10–460 µm and approximately 170 µm in length relatively short fiber geometries [81,98]. The integration of process residues in wood-based panels could therefore be a challenge.

## 3. Utilization of Alternative Furnish Materials for Panel Manufacturing

The application of NWLM and other alternative furnish materials solely or mixed with wood fibers or chips in panel production has been extensively studied during the last decades. Various studies are summarized in Table 5, Table 6, Table 7 and Table 8, where these materials were used as raw materials in fiberboard or particleboard, in combination with another material, as well as the type and quantity of adhesives. Tests in which NWLM or ARs were only used as a filler or as an adhesive component were not considered. The produced panels were evaluated for their mechanical and physical properties, i.e., internal bond (IB), modulus of elasticity (MOE), modulus of rupture (MOR), thickness swelling (TS), and water absorption (WA). 

In most cases, however, only some of the properties were examined in the various studies. Since IB and MOR were predominantly tested and are included in the standard and industry requirements for the application of panels in the dry interior, IB and MOR values are presented in the tables. In addition, it is highlighted whether they meet the minimum requirements of the European Norm (EN) 622–3: 2004 for fiberboard and EN 312: 2003 for particleboard. The panel thickness and density were considered in each case. Unless otherwise noted, material mixes are at a 1:1 weight ratio.

In general, it can be stated that the combinations of NWLM (Table 5) or AR (Table 6, Table 7 and Table 8) with a wooden material usually show better properties than panels without wood content. Up to a proportion of approximately 30% of wood substitutes, the required strength properties are usually achieved. Beyond that level, the properties decrease significantly. Compared to AR, panels containing NWLM achieve higher MOR values, which could be due to the longer fibers. 

Tröger et al. [80] reported that the addition of long flax fibers by 20% in the surface layer (SL) increased the bending properties and decreased the IB values in three-layer particleboards. Papadopoulos and Hague [106] mixed industrial wood chips and flax fibers (0%, 10%, and 30%) in single-layer particleboards by using a 13% urea-formaldehyde (UF) resin binder. Panels with a 30% flax share met the European Standard of P3 particleboard requirements in terms of MOR, IB, and TS. However, the mechanical strength of panels made from 100% wood was always higher. Particleboards made with 100% flax fibers had an insufficient IB strength but an acceptable MOR for P2 boards. The authors attribute the low IB to the relatively thin cell walls of flax. 

Bamboo particles as raw material for particleboards bond with 8% UF resin were examined by Hiziroglu et al. [103]. The single-layer panels of 100% bamboo, or combined with rice stalks or *Eucalyptus*, showed acceptable strength to meet the standard requirements of EN 312:2003. Nikvash et al. [107] investigated three-layer particleboards with different combinations of industrial wood chips and bagasse, canola, or hemp in the core layer (CL). A UF adhesive dosing of 10% in the surface layer and 8% in the core layer was used as a binder in all panels. The results were compared with the control boards made from 100% industrial wood chips. 

**Figure 3:**
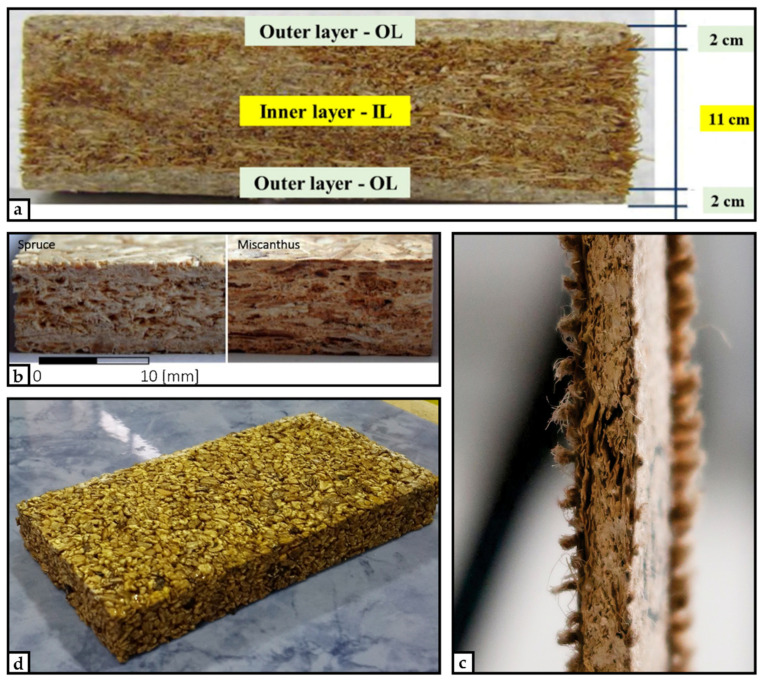
(**a**) three-layer particleboard with green coconut fibers in the outer layer and sugarcane bagasse in the inner layer [155], (**b**) spruce vs. miscanthus single-layer particleboards [156], (**c**) three-layer particleboard with rice husk core and a jute surface layer bonded by soybean protein [157], the (**d**) olive stone particleboard [148].

It was shown that particleboards with 50% bagasse or hemp in the core layer fulfilled the standard requirements for IB, MOR, and TS. The IB strength of the panels with 50% canola share was considerably low. However, the panels with a 30% canola share also met the IB requirements (EN 312:2003). Three-layer particleboards with bagasse in the core and coconut fiber in the surface layer bonded with 15% (SL) and 12% (CL) polyurethane (PUR) resin were examined by Fiorelli et al. [155]. The boards met all the ANSI A20.1-1999 requirements for interior particleboards (Figure 3a).

Akgül and Çamlibel [102] and Yushada et al. [110] considered the use of the rather unusual non-wood lignocellulosic materials rhododendron and seaweed for the production of MDF (medium density fiberboard) and particleboards. MDF panels produced with 100% rhododendron fibers and 11% UF met the minimum requirements of IB, MOR, and MOE for indoor application according to the EN 622-3:2004 standard. 

Single-layer particleboards produced with seaweed and different level of adhesive loads (25%, 28%, and 30% UF) showed acceptable IB strength by reaching the standard level (Japanese Industrial Standard JIS A 5908). In comparison, the measured MOR and MOE values were significantly below the minimum requirements of the standard, even at the highest adhesive load of 30% UF. The low values could be explained by incomplete curing of the UF adhesive with the seaweed particles [110]. 

Balducci et al. [109] studied the performance of one-layer particleboards made with miscanthus and 6% polymeric diphenylmethane diisocyanate (pMDI) or an unknown amount of UF resin. The pMDI-bonded boards met the standard for all properties (IB, MOE, MOE), while the UF-bonded ones did not meet the minimum requirements for IB (EN 312:2003). Compared to single-layer boards, three-layer particleboards bonded with an undefined amount of UF adhesive had a lower IB but higher MOR, MOE, and TS values. An example of one-layer particleboards from miscanthus compared to a spruce particleboard is given in Figure 3b.

The research on using grow care residues for panel manufacturing is rather scarce (Table 6). Three-layer particleboards prepared by replacement of wood chips with 50% vine pruning particles in the core layer showed comparable mechanical properties to panels made with 100% wood chips. Those panels used 12% and 8% UF resin in the surface and core layers, respectively. A negative effect on the mechanical properties of the panels was observed with the increased content of vine pruning particles. Similar performance reduction was also observed when vine pruning particles were used in one-layer particleboards [54]. 

According to the authors, the reduction is due to the lower length to thickness ratio compared to wood particles, as well as the certain amount of pith particles in the material. With single-layer particleboards from vine pruning waste and 9% UF, Ferrandez-Villena et al. [116] showed that it is even possible to reach the minimum requirements for furniture manufacturing. However, with a high panel density of approximately 865 kgm^−3^. Nemli et al. [113] investigated different versions of three-layer particleboards with kiwi pruning particles in the core layer. An industrial UF resin was used with 11% and 8% in surface and core layers, respectively. 

An increase of kiwi pruning particles in the core layer negatively affected the panel properties. The reduction is also justified by the proportion of pith and bark in the kiwi pruning material. Panels containing up to 50% kiwi pruning particles exceeded the minimum requirements of MOR according to EN 312:2003 for general purposes. The mechanical strength of the panels was improved slightly by increasing the adhesive content by 1% for each panel version. 

Ayrilmis et al. [111] used ground pinecones from 0 to 50% to replace wood fibers in MDF panels bonded with 10% of UF adhesive. The authors reported that the water resistance of MDF panels was improved by increasing the pinecone content up to 10%. The mechanical properties of the MDF panels, however, decreased with increasing the amount of pinecone. It was assumed that the pinecone material acted more as a formaldehyde scavenger than as a strength provider since the formaldehyde emissions decreased with increasing the pinecone content. 

In the case of harvest residues, particleboard panels were studied more than any other panel type (Table 7). Harvest residues from castor [158], cotton [124], eggplant [128], pepper [132], canola [83], rice [44], sorghum [159], sunflower [135], tomato [138], wheat [120], and mustard stalks [129] processing were used in a series of combinations with industrial wood chips and UF adhesive for the production of single and three-layer particleboards. A maximum of 30% replacement of wood chips with canola stalks in the core layer of three-layer particleboards, with 10% UF resin in the surface layer and 8% in the core layer, showed comparable IB strength to the standard requirements [107]. 

Grigoriou and Ntalos [158] quoted that a 50% share of castor stalks was the optimum amount to reach an acceptable MOR and IB strength in single-layer particleboard panels using 8% UF adhesive. Application of corn, triticale, or rye stalks in the surface layer of three-layer particleboard panels together with 4% pMDI resulted in higher MOR and MOE than the control panels prepared with sole pine chips. Panels with reed stalks in the surface layer, on the other hand, had lower MOR and IB than the controls. All prepared panels fulfilled the standard requirements for MOR and MOE; however, only the ones made with corn stalks met the minimum requirements for IB strength [134]. 

Compared to the control panels, TS was lower in all experimental panels. Panels made from rye had 15% less TS than controls. The authors reported that the reduction in TS of the particleboards could be attributed to the hydrophobic nature of the rye stalks. Single-layer particleboard panels made with different mixing ratios of hardwood and pepper stalk particles and 8% of UF resin, showed decreasing mechanical properties with an increased amount of pepper stalk particles [132]. According to Khristova et al. [135], and Grigoriou and Ntalos [158], the utilization of pith from sunflower stalks is not recommended as it negatively affects the mechanical strength and water-related properties of particleboards. 

Palm tree wood was used with UF adhesive for the production of particleboard and plywood panels [127,160], and the results showed that three-layer particleboards made from 100% palm particles, and a respective adhesive load of 11% and 9% in the surface and core layers, met the minimum requirements for interior fitments in IB, MOR, and TS (EN 312:2003). Hashim et al. [130] studied the performance of binderless single-layer particleboards made with oil palm wood and reported that the panels achieved the minimum requirements for IB but not for MOR according to the Japanese Industrial Standard (JIS A-5908 Type-8). The low MOR is explained by the lack of an adhesive.

Among the side streams from the agricultural industry, process residues and industrial food residues have received the most attention for panel production recently (Table 8). Pirayesh and Khazaeian [61] reported that three-layer particleboards manufactured with almond husks, 9% UF resin in the core layer, and 11% in the surface layer, met the minimum requirements for MOR and IB (EN 312:2003) at a maximum level of 30% replacement of wood chips. 

With a higher proportion of husks, the generally poorer bonding of the resin and the almond husks lead to significantly reduced mechanical properties. 30% was also given as the highest proportion in fiberboard panels with hazelnut husk [141] and particleboard panels with sugar beet pulp [150]. Binderless single-layer particleboards from almond husk pressed at low temperature (120 °C) for 30 min met the minimum requirements for panels for interior use. The achieved strength has been attributed to the high sugar content. After such long pressing time, the sugar acted as a binder between the particles [143]. Guler et al. [63] studied the performance of three-layer particleboards using peanut husks and UF resin (10% surface layer and 8% core layer). 

They suggested 25% peanut husks as the optimum level to achieve the standard requirements for boards in interior applications. The MOR and MOE values, in particular, decreased with a higher proportion of peanut husks. The panels with 100% peanut husks, on the other hand, showed lower TS than the ones with 25% husks. High density (>940 kgm^−3^) one-layer particleboards made with 15%, 30%, and 100% oat husks and 10% polyurethane resin reached the acceptable level of the EN 312:2003 standard for general purpose in MOR and IB [161]. 

Recently, Farag et al. [148] used olive stones together with an unsaturated polyester liquid resin for preparing single-layer particleboard panels, and they found that the panels fulfilled the MOR requirements for general purpose (EN 312:2003) at 20% adhesive load (Figure 3d). However, the maximum permitted values mentioned in the EN 312:2003 standard for the wet condition in TS were slightly exceeded. Single-layer particleboard panels from rice husks and 8% UF were tested by Melo et al. [105]. The rice husk panels showed significantly lower MOR, MOE, IB, and higher TS than the reference panels from industrial wood particles. 

The authors report that one reason may be the cylindrical and hollow structure of the hole rice husk particles, which could act as a barrier during gluing. Likewise, a lower permeability of the husks for the resin could have a negative effect on an even distribution of the adhesive. Faria et al. [149] investigated three-layer particleboards from *Eucalyptus* wood, different proportion of soybean husks in the CL and 10% UF. Panels with 100% soybean husks in the core layer did not meet any standard requirements. 

However, a high MOR was observed with a 1:1 ratio of *Eucalyptus* wood and soybean husks. The MOR increase was attributed to various factors, such as a higher interaction of the particles due to the increase of the compression ratio and better adhesive distribution on the particles. A combination of raw materials from non-wood lignocellulosic and agricultural sources was also used for panel preparation [160].

Khedari et al. [62] reported particleboards with low thermal conductivity using coconut coir and durian peel and combinations thereof, bonded with 12% UF resin. They found that a 90:10 mix ratio of coconut coir and durian peel was the optimum to fulfill the minimum requirements for IB values according to the Japanese Industrial Standard (JIS A-5908 Type-8). 

Nicolao et al. [157] developed particleboard from a combination of rice husks and jute fibers. The three-layer panel consisted of a rice husk core and different numbers of jute fiber surface layers bonded with 10% soybean protein adhesive (Figure 3c). With MOR from 12.6 to 27.9 Nmm^−2^, the bending properties improved with an increased number of jute surface layers.

In addition to the classic panel types, fiberboard, and particleboard, investigations were also conducted with plywood or special panels but to a lesser extent. Abdul Khalil et al. [160] tested five-layer plywood with UF or PF from oil palm wood (500 gm^−2^), as well as five-layer hybrid plywood with two layers consisting of oil palm empty fruit bunch fibers. The hybrid plywood achieved higher MOR and MOE than the oil palm wood plywood. It was attributed to the higher density of the hybrid panel.

The studies described in Table 5, Table 6, Table 7 and Table 8 show that NWLM and ARs, especially in fiberboards and particleboards, were extensively tested and the requirements were met in many cases. In particular, NWLM benefit from their long fibers in the panels. Grow care residues have been little studied thus far, and their integration into panels also negatively influences the bending behavior. The much-noticed harvest residues are generally well suited for both fiberboard and particleboard. They perform particularly well in combination with wooden material. Various husk types of process residues could not achieve sufficient bending strength values. Other process residues, such as coconut coir or sugarcane bagasse, appear suitable as raw materials for panels.

## 4. Panel Manufacturing Parameters

The performance of the panels prepared from alternative furnish raw materials are highly influenced by production parameters, including adhesive type and ratio, panel density, and pressing factors (speed and press temperature). Previous studies have shown a direct relation between the panel’s adhesive type and ratio and mechanical properties [91,162].

Papadopoulos et al. [163] revealed that the mechanical properties of bamboo particleboards increased with increasing the UF adhesive loads from 10% to 14%. Similar results were reported for UF-bonded particleboards made with cotton stalks [125]. UF, phenol-formaldehyde (PF), and melamine urea-formaldehyde (MUF) adhesives at 10% and 8% load were used for the manufacturing of three-layer particleboards with hazelnut husks, and the results illustrated identical mechanical properties for panels bonded within UF and PF, and lower MOR and IB values for those with MUF [146]. Barbu et al. [164] compared single-layer particleboards from walnut and hazelnut husks bonded with 10% MUF or PUR adhesive. 

Both panels with PUR adhesive illustrated higher bending properties (MOR and MOE) and lowered TS values than the MUF-bonded panels. The compatibility of various alternative furnish materials with conventional adhesive systems is rather challenging. For instance, the curing behavior of a standard UF adhesive in the hot press depends not only on the hardener type and the pressing temperature but also on the pH value of the raw material [165]. The presence of a high amount of wax and silica in stalks and husks cause poor interactions at the interfaces between the adhesive and the substrates. It also hampers the proper poly-condensation of the MUF adhesive, which results in weak bond lines [33]. 

Apart from the common UF and MUF adhesives, other adhesive types were also investigated for manufacturing panels from NWLM and ARs, such as pMDI [33], PF [166], bio-based systems [167], natural rubber [168] and soybean flour [139]. When using pMDI, the panel requirements are met in almost all studies (Table 5, Table 7 and Table 8). Pan et al. [169] evaluated the performance of the single-layer particleboards made with rice stalks and a 4% adhesive mixture of pMDI and rice bran. The authors suggested that 20% of the adhesive can be replaced by rice bran while achieving a comparable mechanical strength to the control panel. Single-layer rice stalk particleboards with UF and corn starch as adhesive were compared by Hussein et al. [170]. 

With 10% adhesive load in each case, MOR and IB were significantly lower with corn starch bonded panels than with UF. Methylene diphenyl diisocyanate (MDI), UF, soybean protein isolate (SPI), and defatted soybean flour (SF) based adhesive systems were compared in single-layer wheat stalk particleboards [139]. The mechanical properties (MOR, MOE, and IB) of the panels prepared by 8% UF, 10% SPI, and 15% SF were identical or inferior to the ones manufactured with 4% MDI. 

Single-layer particleboards prepared with corn stover and 10% soy-based adhesive reached the minimum requirements for the bending properties (MOR, MOE) according to American National Standards Institute (ANSI) but not for IB [145]. Battegazzore et al. [167] evaluated the bending properties of fiberboards made with hemp fibers and particleboards with rice husks. Both panel types were bonded with corn starch (37.5% for hemp and 50% for rice husks) and were formed through a wet process. The results showed that both panel types achieved the minimum requirements for the MOR (EN 312:2003).

It is well known that the mechanical properties of wood-based panels are directly related to their density [171,172]. The density of a wood-based panel usually correlates linearly with its mechanical and physical properties. One reason is the increased contact area of the particles or fibers covered by the adhesive. A higher density also allows the adhesive to spread more widely but compromises heat transfer during the pressing process [173]. 

The property can also be transferred to panels from NWLM and ARs [120]. Since the materials generally have a lower density than wood (Table 4), the density of the final panels can also be lower. Previous studies on panels with alternative materials reported density values ranging from 400 [124] to 780 kgm^−3^ [128,138], depending on the material type used. Many studies have though focused on panel densities of about 700 kgm-^3^ [110,125,136,174]. Although the density of raw materials plays a significant role in defining the final density of the panels, the panel density can also be adjusted by other manufacturing parameters, i.e., compression ratio, water content, press temperature, pressing schedule, or adhesive load [175], which may increase the cost of the final product.

The pressing temperature is an essential factor that influences the performance of the panels by providing the thermal energy for curing the adhesive and mechanical compression force to consolidate the mat [176]. The effect of pressing temperature varies with the density of raw material and panel type, as the higher density panels have higher maximum core temperatures due to their capability to build higher internal gas pressure [177]. Binderless particleboards prepared with oil palm trees showed higher mechanical properties by increasing the press temperature [130]. 

The MOE, IB, and TS of MDF panels made from corn stalks and 10% UF adhesive were improved by increasing the press temperatures from 170 to 180 °C, while a negative effect was observed with further increasing the pressing temperature to 190 °C. MDF panels produced with cotton stalks and 10% UF adhesive demonstrated higher bending properties and lower TS values with increasing pressing temperatures from 170 to 190 °C, while opposite results were obtained for IB strength [118]. Nogueira et al. [178] tested three-layer particleboards from sugarcane bagasse and waste plastic bags. A reduction in TS and WA could be observed as the press temperature increased from 160 to 220 °C.

## 5. Material Processing and Pretreatment

In addition to the type of raw materials and manufacturing parameters, the processing and pretreatment affect the properties of the final panel. The raw materials from non-wood lignocellulosic and agricultural sources have been mainly processed mechanically and some were chemically pretreated to create evenly sized particles or to improve their performance in final panels [72,126,139]. The mechanical processing of NWLMs and ARs, such as canola stalks, with a hammermill, has often been used to prepare the raw material [122]. Ndazi et al. [179] produced single-layer particleboards with ground (8 mm sieve) and untreated rice husks and 15% of PF. The results showed that the mechanical properties of the panels decreased by grinding. 

Many factors can be varied in fiber production for fiberboard raw material. Zawawi et al. [180] investigated the influence of refining conditions, pressure and temperature on oil palm fruit husk’s fiber and fiberboard properties. It was found that higher pressure and a higher temperature in the refiner ultimately led to increasing MOE, MOR, IB and reduced water absorption. Overly aggressive refining conditions, however, produced shorter fiber lengths and consequently reduced the fiberboards’ physical and mechanical properties. 

Chemical pretreatments of raw materials are performed to optimize the bonding capability of the particles and the adhesive. Due to the increase in reactive hydroxide (OH) groups during an alkali treatment, the binding of the raw material and the adhesive improves [181,182]. The hydrophilic nature of raw materials can also reduce mechanical properties due to water absorption and a reduction of the water resistance. Acetylation can increase the hydrophobicity of a raw material, which leads to less thickness swelling and improved mechanical strength. [183].

Cotton stalks pretreated with 1–5% sodium hydroxide (NaOH) were used to prepare single-layer particleboards in combination with 10% UF adhesive [126]. The NaOH decomposes the lignin and reduces the content of hemicelluloses and extractives. In relative terms, since cellulose is more resistant to NaOH, the proportion increases. In addition, the surface roughened by the NaOH treatment offers better bonding between raw material and adhesive [184]. 

**Figure 4:**
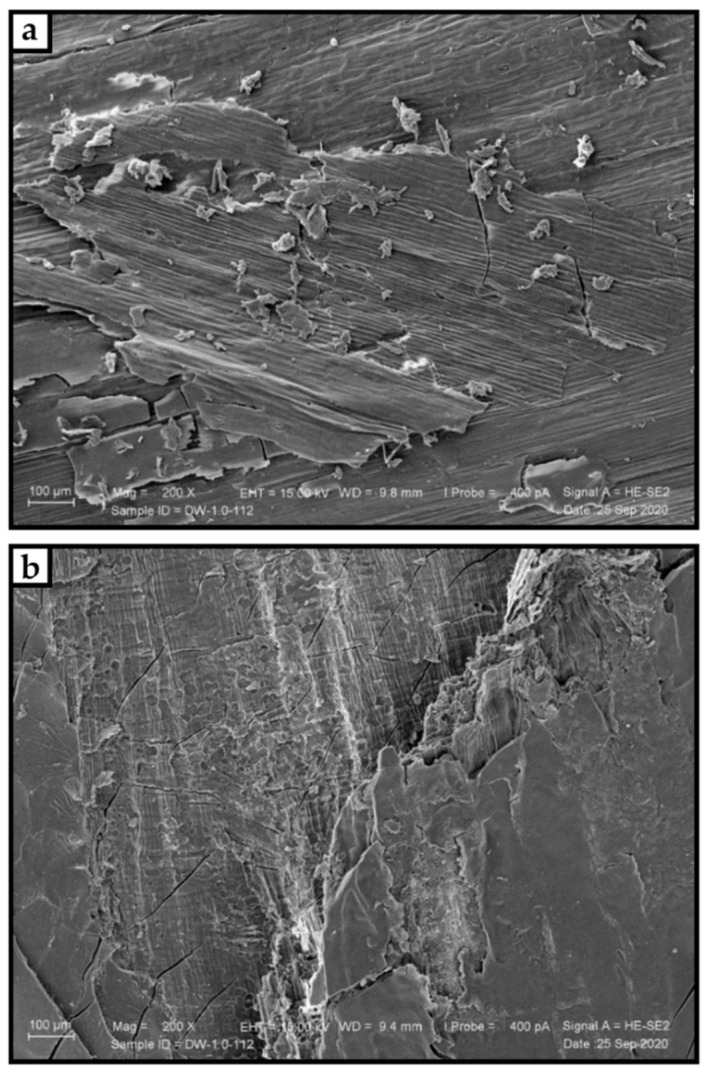
Increase of surface roughness of canola stalks (**a**) for particleboard panels after treatment with sodium hydroxide (**b**) [184].

The results showed that the static bending properties of the panels (MOE and MOR) improved by 1% NaOH treatment, while pretreatment of the cotton stalks at 3% and 5% of NaOH resulted in the strength reduction of the panels. Treatment at a higher concentration degrades the cell wall components stronger and deforms the particle structure, resulting in a reduction in mechanical strength. Figure 4 shows NaOH treated canola stalks with rougher surface than untreated ones. Mo et al. [139] bleached wheat stalks with 3% sodium hypochlorite in a ratio of 1:10 for 30 min at 50 °C. When bleaching lignocellulosic materials, the hydrophobic wax and inorganic components on the surface, such as silica, are removed. 

This increases the wettability of the stalks and the bonding ability of the entire panel with water-based adhesives [185]. The pretreatment led to a significant improvement in mechanical properties (MOE, MOR, and IB) of single-layer particleboards bonded with 8% UF adhesive. Despite the intensification of the hydrophilic nature of the stalks, TS and WA only increased slightly. Among other things, TS is closely related to the bonding quality. As this could be significantly enhanced, less water could penetrate into the panels, and the increased hydrophilicity had little effect [139]. 

To optimize the curing behavior of UF resin in fiberboards made with wheat stalks, Halvarsson et al. [120] pretreated the stalks with a 10% sulfuric acid solution to decrease the pH value below 6 before fiber refining. However, no significant changes in the mechanical properties of the panels were determined. Ciannamea et al. [72] compared the effects of alkaline and alkaline-oxidation pretreatments on the performance of single-layer particleboards made with rice husks using 10% of a modified soybean protein adhesive. 

The authors reported that the two-phase pretreatment, NaOH followed by a hydrogen peroxide treatment, resulted in higher mechanical strength and lower water resistance in comparison with panels prepared by only alkaline pre-treated rice husks and also with those with untreated husks. This is due to the reduction of lipids and waxes after the peroxide treatment, thus, allowing a better bonding of the raw material and the adhesive. Single-layer particleboard panels made from corn stalks and 10% soy-based resin were tested by Ren et al. [145]. 

The corn stalks were fermented in a process similar to the common procedure used in agriculture. Crops were stored under anaerobic conditions to inhibit undesirable microbial growth and prevent deterioration. The boards from corn stalks, fermented for 21 days, showed improved MOR and MOE as well as significantly increased IB values. 

TS and WA were also noticeably reduced. However, with a longer fermentation than 21 days, all mechanical and physical properties deteriorated again. The reason was assumed to be the interaction of several biological factors. The surface structure may have increased due to the hydrolysis of carbohydrates, and a micro biofilm may act as an adhesive.

## 6. Conclusions and Future Scopes

Lignocellulosic materials from non-wood and agricultural sources represent a potential alternative choice to wood materials for wood-based panel manufacturing. These materials are derived from renewable sources and can be used as a partial or entire replacement for wood chips and fibers. One advantage of most of these materials for easier integration in industrial manufacturing processes of wood panels is their similar nature to wood materials in terms of chemical composition and fiber morphology. 

However, these alternative raw materials suffer from several different issues that prevent their application in industry. Some NWLMs have to be cultivated separately, and they are in direct economic competition with food agriculture and its land use. Economically practical use of grow care residues currently appears rather unlikely for wood-based panels as they result in low-performing panels. The different stalk types of harvest residues usually show shorter fiber lengths and a high extractive content, which can primarily affect the bonding quality and affinity to the adhesive in the panel. 

It should be noted that laboratory panels from alternative panels were tested for their use in interior furniture, and their strength performance is sufficient for load-bearing purposes. A great challenge is that the agricultural harvest is only performed seasonally, and therefore no continuous flow of raw materials can be guaranteed. Storage capacities would be required to ensure constant production with seasonal harvest residues. This would result in high costs, and the influence of long-term storage on the raw material should be examined beforehand. 

The bulk density of most alternative materials is low, thereby, making their handling more expensive than wood [131] and contributing to high logistic costs for their transportation [30]. In addition, it appears that there is an upper limit on the wood replacement ratio. If the proportion of an alternative raw material to wood is higher than 30% in combination with traditional UF resin, the mechanical properties deteriorate significantly. The thickness swelling and water absorption of experimental panels are also relatively high. 

However, the use of alternative synthetic adhesives, especially pMDI, shows that panels from 100% agricultural residues can also fulfill the requirements. A life cycle assessment (LCA) for sugarcane bagasse added in particleboard demonstrated that the agricultural residue can replace the traditional wood as a raw material due to its better environmental performance. The required mechanical properties of panels can be achieved, comparatively less land is occupied, and further material use reduces abiotic depletion and ecotoxicity [186].

The selection of new raw materials should focus on plants grown for various purposes and have an appropriate structural composition. There should be no competition for the use of the residues, their price should be low, and sufficient quantities should be available. The industrial production of wood-based panels is a process that has been optimized over decades, and a modification of the handling and processing of raw materials requires long-term optimization processes. Therefore, initially, small volume niche products should be considered as more feasible panel types for those materials. 

The increased production volume of fiberboards in the last few decades provides good future opportunities. Since the production of fiberboards has increased considerably (Figure 1), the high availability of hemp and flax fibers provides an advantage for using these materials. Compared to wood, the fibers are longer and have a higher cellulose content. The use of 100% flax fibers in panels was proven to be sufficient to produce mechanically robust panels at a laboratory scale, though further optimization is needed to improve their internal bond strength. 

Trunks of wood species from which the fruit is used commercially can serve as a substitute raw material. For example, large amounts of harvest residues from oil palm production can be used more effectively. Panels made from oil palm wood, which is less tied to seasonal harvest, have mostly shown adequate mechanical properties. Harvest residues are suitable as raw materials because they have an enormously high production potential. When harvesting wheat and rice, more than the same amounts of stalk residues accumulate. 

In addition, the tested panels achieved acceptable properties with a suitable adhesive. An advantage of the process residues is that, typically, they are already integrated into an industrial material flow system, e.g., flour production, which can reduce the logistical effort for panel manufacturing. For example, oat and hazelnut husks have similar chemical compositions. 

Furthermore, the panels produced can meet the minimum requirements for mechanical strength. In addition, the materials have already been removed from the ecosystem, and using them as raw material for panels adds value and enhances sustainability compared to thermal incineration only. An integration of husks for special panel types with adapted requirements should be considered. Consequently, selected raw materials from the various categories can be used in wood-based panels. The first thing to consider is their local availability and the intended use. Focus should be given to an appropriate type of adhesive and dosing. 

In particular, alternatives to UF, such as pMDI or PF, have proven that panels made entirely of agricultural residues can meet the requirements. It should be kept in mind that additional costs might occur for the pretreatment of some raw materials as a necessary or optional approach to improve the panel performance. The information gathered in this review provides the set of current knowledge in this research field. It identifies promising alternative raw materials and their challenges in replacing partially or entirely standard wood materials for more sustainable wood-based panel production. 

Finally, it should be noted that this review has its limitations. No definite conclusions can be made on ideal alternative materials from agro-industry to substitute wood in wood-based panels as there are many factors to consider. Further investigations should focus on regional availability of such materials and demand for specific product types. Then, suitable alternative materials could be identified more clearly. The search for new raw materials could also be expanded, such as to include raw materials from short-rotation plantations or plantations in general. The focus of this work has been on fiberboard and particleboard panels. The wide range of applications of non-wood lignocellulosic materials and agricultural residues in other panel types, such as wood–plastic composites, should also be considered in the future.

## Figures and Tables

**Table 1:** Forest land vs. cropland area in the world in 2019 (million km^2^, [19]).

Area	Forest Land	Cropland
Africa	6.41	2.76
Asia	6.2	5.90
Europe	10.17	2.89
North America	6.57	1.99
Oceania	1.85	0.33
South America	8.46	1.32

**Table 2:** Residue-to-crop ratio and amount of crop production in 2018 (million tons, [19]).

Crop	Residue-to-Crop Ratio			Production		Stalks		Husks		Leaves
	Stalks	Husks	Leaves	World	Europe	World	Europe	World	Europe	World
Sugarcane	0.26	-	0.2	1907.0	2.3	495.8	0.6	-	-	381.4
Corn	1.96	0.22	-	1147.6	128.6	2249.3	252.0	252.5	28.3	-
Rice	1.33	0.25	-	782.0	4.0	1040.1	5.4	195.5	1.0	-
Wheat	1.28	-	-	734.0	242.1	939.6	309.9	-	-	-
Potato	0.25	-	-	368.2	105.2	92.0	26.3	-	-	-
Soybean	1.53	1.09	-	348.7	12.1	533.5	18.4	380.1	13.1	-
Sugar beet	0.25	-	-	274.9	185.1	68.7	46.3	-	-	-
Oil palm	0.31	-	2.6	272.1	0.1	84.3	-	-	-	707.3
Coconut	-	0.49	0.47	61.9	-	-	-	30.3	-	29.1
Sorghum	2.44	-	-	59.3	1.1	144.8	2.6	-	-	-
Groundnut	-	0.47	-	46.0	-	-	-	21.6	-	-
Cotton	3.4	0.26	-	41.2	0.5	140.0	1.9	10.7	0.1	-
Millet	2.54	-	-	31.0	0.4	78.8	1.0	-	-	-
Oat	1.42	-	-	23.1	13.5	32.7	19.2	-	-	-
Barley	1.35	-	-	14.1	83.1	19.1	112.2	-	-	-
Rye	1.61	-	-	11.3	9.1	18.2	14.7	-	-	-
Coffee	-	1.32	-	10.3	-	-	-	13.6	-	-
Cacao	-	1.5	-	5.3	-	-	-	7.9	-	-
Total				6137.9	787.3	5936.9	810.6	912.2	42.6	1117.8

**Table 5:** NWLM used for panel production with information on whether MOR and IB meet (**✓**) or not (**✕**) the standard requirements (fiberboard EN 622-3:2004; particleboard EN 312:2003).

Panel Type	Materials		Resin Type SL; CL (%)		MOR (Nmm^−2^)		IB (Nmm^−2^)		References
Fiberboard	Bamboo	Bagasse fiber	UF	4	12	**✕**	1.4	**✓**	[74]
	Kenaf	-	-	-	18 *	**✓**	0.2 *	-	[82]
	Kenaf	-	UF	10	29.14	**✓**	0.33	**✓**	[101]
	Rhododendron	-	UF	11	40 *	**✓**	0.63 *	**✓**	[102]
	Rhododendron	ind. wood fibers	UF	11	32 *	**✓**	0.60 *	**✓**	[102]
									
Particleboard	Bamboo	-	UF	8	22.57	**✓**	1.61	**✓**	[103]
	Bamboo	*Eucalyptus*	UF	8	25.25	**✓**	1.62	**✓**	[103]
	Bamboo	Rice stalks	UF	8	14.36	**✓**	0.1	**✕**	[103]
	Bamboo	-	UF	8	13.44	**✓**	0.32	**✓**	[104]
	Bamboo	-	PF	8	13.6	**✓**	0.26	**✕**	[104]
	Bamboo	*Pinus taeda*	PF	8	17.68	**✓**	0.4	**✓**	[104]
	Bamboo	-	UF	8	11.25	**✕**	0.22	**✕**	[105]
	Bamboo	*Eucalyptus*	UF	8	12.79	**✓**	0.22	**✕**	[105]
	Flax	-	UF	13	11.72	**✓**	0.09	**✕**	[106]
	Flax	ind. wood chips	UF	13	13.22	**✓**	0.43	**✓**	[106]
	Hemp	-	UF	10; 8	16 *	**✓**	0.78 *	**✓**	[107]
	Hemp	ind. wood chips	UF	10; 8	16 *	**✓**	0.78 *	**✓**	[107]
	Jose wheat grass	-	pMDI	4	19.6	**✓**	-	-	[108]
	Kenaf	-	UF	8	12.88	**✓**	0.86	**✓**	[101]
	Miscanthus	-	pMDI	6	24.2	**✓**	0.11	**✕**	[80]
	Miscanthus	-	UF	12	11	**✕**	0.67	**✓**	[80]
	Miscanthus	-	pMDI	6	5.7	**✕**	0.23	**✕**	[109]
	Seaweed		UF	25	2.6 *	✕	5.8 *	✓	[110]

* derived from figure, CL (core layer), ind. (industrial), PF (phenol-formaldehyde), pMDI (polymeric diphenylmethane diisocyanate), SL (surface layer), and UF (urea-formaldehyde).

**Table 6:** Grow care residues used for panel production with information on whether MOR and IB meet (**✓**) or not (**✕**) the standard requirements (fiberboard EN 622-3:2004; particleboard EN 312:2003).

Panel Type	Materials		Resin Type SL; CL (%)		MOR (Nmm^−2^)		IB (Nmm^−2^)		References
Fiberboard	Pinecone	ind. Wood fiber	UF	10	13.3		0.4	-	[111]
									
Particleboard	Grass clipping	-	UF	12	4.19	**✕**	0.08	**✕**	[112]
	Grass clipping	Eucalyptus chips	UF	12	8.39	**✕**	0.189	**✕**	[112]
	Kiwi pruning	-	UF	10; 8	8.42	**✕**	0.527	**✓**	[113]
	Kiwi pruning	ind. wood chips	UF	10; 8	10.47	**✕**	0.555	**✓**	[113]
	Needle litter	-	UF	12	6.83	**✕**	0.152	**✕**	[114]
	Needle litter	ind. wood chips	UF	12	9.15	**✕**	0.208	**✕**	[114]
	Vine pruning	-	UF	8	8.5	**✕**	0.69	**✓**	[54]
	Vine pruning	-	UF	8	3.75	**✕**	0.3	**✓**	[115]
	Vine pruning	-	UF	9	13.6	**✓**	1.32	**✓**	[116]
	Vine pruning	-	UF	10	4.17	**✕**	0.33	**✓**	[115]
	Vine pruning	ind. wood chips	UF	8	14	**✓**	0.84	**✓**	[54]
	Yerba mata pruning	-	UF	8	9.6	**✕**	1.05	**✓**	[117]
	Yerba mata pruning	ind. wood chips	UF	8	14.5	✓	1.28	✓	[117]

CL (core layer), ind. (industrial), SL (surface layer), and UF (urea-formaldehyde).

**Table 7:** Harvest residues used for panel production with information on whether MOR and IB meet (**✓**) or not (**✕**) the standard requirements (fiberboard EN 622-3:2004; particleboard EN 312:2003).

Panel Type	Materials		Resin Type SL; CL (%)		MOR (Nmm^−2^)		IB (Nmm^−2^)		References
Fiberboard	Canola stalks	-	UF	9	18.95	**✓**	0.414	**✓**	[83]
	Corn stalks	-	UF	10	22.26	**✓**	0.415	**✓**	[118]
	Rice stalks	-	pMDI	3	26 *	**✓**	1.3	**✓**	[119]
	Wheat stalks	-	UMF	14	31 *	**✓**	0.7 *	**✓**	[120]
									
Particleboard	Canola stalks	-	MUPF	8	11.1	**✕**	0.31	**✓**	[121]
	Canola stalks	-	pMDI	8	14.7	**✓**	0.82	**✓**	[121]
	Canola stalks (CL)	-	UF	10; 8	13 *	**✓**	0.12 *	**✕**	[107]
	Canola stalks	-	UF	8	11	**✕**	0.28	**✓**	[121]
	Canola stalks (CL)	ind. wood chips	UF	10; 8	14.5 *	**✓**	0.21 *	**✓**	[107]
	Canola stalks	ind. wood chips	UF	12	9.1	**✕**	0.25	**✓**	[122]
	Coconut wood	-	EMDI	4	14.21	**✓**	0.54	**✓**	[121,123]
	Cotton stalks	-	PF	12; 10	17.95	**✓**	0.591	**✓**	[107,124]
	Cotton stalks	-	UF	10	14.6	**✓**	0.6	**✓**	[122,125]
	Cotton stalks	-	UF	10	8.1	**✕**	0.34	**✓**	[123,126]
	Date palm	-	UF	11; 9	18.14	**✓**	0.67	**✓**	[127]
	Eggplant stalks	-	MUF	12; 10	13.2	**✓**	0.966	**✓**	[128]
	Eggplant stalks	-	UF	12; 10	13.14	**✓**	0.5	**✓**	[128]
	Mustard stalks	-	UF	12	14.5	**✓**	0.29	**✓**	[129]
	Mustard stalks	ind. wood chips	UF	12	14.7	**✓**	0.59	**✓**	[129]
	Oil palm wood	-	-	-	4.9 *	**✕**	0.37 *	**✓**	[130]
	Pepper stalks	-	UF	12; 10	12.32	**✓**	0.83	**✓**	[131]
	Pepper stalks	-	UF	8	12.2	**✕**	0.61	**✓**	[132]
	Pepper stalks	trop. hardwood	UF	8	14.2	**✓**	0.71	**✓**	[132]
	Primrose stalks	pine chips (SL)	MUPF	12; 10	14.3	**✓**	0.57	**✓**	[133]
	Primrose stalks	pine chips (SL)	pMDI	8; 6	19	**✓**	0.9	**✓**	[133]
	Primrose stalks	pine chips (SL)	UF	12; 10	15.7	**✓**	0.41	**✓**	[133]
	Reed stalks	ind. wood chips	pMDI	6; 4	14.1 *	**✓**	0.31 *	**✓**	[134]
	Rice stalks	-	pMDI	4	14 *	**✓**	0.46 *	**✓**	[44]
	Rice stalks	-	UF	12	7 *	**✕**	0.15 *	**✕**	[44]
	Rye stalks	ind. wood chips	pMDI	6; 4	29 *	**✓**	0.32 *	**✓**	[134]
	Sorghum stalks	ind. wood chips	UF	8	10 *	**✕**	0.61 *	**✓**	[91]
	Sunflower stalks	-	PF	12	10.28	**✕**	0.16	**✕**	[135]
	Sunflower stalks	-	UF	11; 9	15.65	**✓**	0.46	**✓**	[136]
	Sunflower stalks	ind. wood chips	PF	12	6.98	**✕**	0.11	**✕**	[135]
	Sunflower stalks	pine chips	UF	11; 9	18.74	**✓**	0.58	**✓**	[137]
	Sunflower stalks	*Popolus alba* L.	UF	11; 9	22.03	**✓**	0.51	**✓**	[136]
	Tomato stalks	-	MUF	12; 10	12.75	**✓**	0.69	**✓**	[138]
	Tomato stalks	-	UF	12; 10	10.89	**✕**	0.53	**✓**	[138]
	Tomato stalks	-	UF	12	12.5 *	**✓**	0.38 *	**✓**	[94]
	Triticale stalks	ind. wood chips	pMDI	6; 4	25 *	**✓**	0.32 *	**✓**	[134]
	Wheat stalks	-	MDI	4	11.45	**✕**	0.64	**✓**	[139]
	Wheat stalks	-	PF	10	16.9	**✓**	0.68	**✓**	[140]
	Wheat stalks	-	UF	8	3.96	**✕**	0.11	**✕**	[139]

* derived from figure, CL (core layer), EMDI (emulsified diphenylmethane diisocyanate), ind. (industrial), MUF (melamine urea-formaldehyde), MUPF (melamine urea phenol-formaldehyde), PF (phenol-formaldehyde), pMDI (polymeric diphenylmethane diisocyanate), SL (surface layer), and UMF (urea melamine-formaldehyde).

**Table 8:** Process residues used for panel with information on whether MOR and IB meet (**✓**) or not (**✕**) the standard requirements (fiberboard EN 622-3:2004; particleboard EN 312:2003).

Panel Type	Materials		Resin Type SL; CL (%)		MOR (Nmm^−2^)		IB (Nmm^−2^)		References
Fiberboard	Hazelnut husks (30%)	ind. wood fibers	UF	8	13.9	**✓**	0.22	**✓**	[141]
	Oil palm fruit husks	-	PF	6	32.8	**✓**	0.114	**✓**	[142]
	Oil palm fruit husks	-	PF	10	27.2	**✓**	0.24	**✓**	[142]
									
Particleboard	Almond husks	-	-	-	14.01	**✓**	0.90	**✓**	[143]
	Almond husks	-	UF	11; 9	7.41	**✕**	0.27	**✓**	[61]
	Almond husks	ind. wood chips	UF	11; 9	10.2	**✕**	0.36	**✓**	[61]
	Coconut Coir	-	UF	11; 7	15.1	**✓**	0.40 *	**✓**	[144]
	Coconut Coir	pine chips	UF	11; 7	17.5	**✓**	0.32	**✓**	[144]
	Coconut Coir	Durian husks	UF	12	36.8	**✓**	0.3	**✓**	[62]
	Coffee husks	ind. wood chips	MUPF	15	11.9	**✓**	0.34	**✓**	[33]
	Coffee husks	ind. wood chips	pMDI	8	14.1	**✓**	0.6	**✓**	[33]
	Coffee husks	ind. wood chips	UF	15	13.1	**✓**	0.41	**✓**	[33]
	Corn stover	-	soy	10	16.5 *	**✓**	0.8 *	**✓**	[145]
	Hazelnut husks	-	MUF	10; 8	10.1	**✕**	0.39	**✓**	[146]
	Hazelnut husks	-	PF	10; 8	12	**✓**	0.482	**✓**	[146]
	Hazelnut husks	-	UF	10; 8	11.9	**✓**	0.505	**✓**	[146]
	Macadamia husks	-	PU	20	4.3	**✕**	1.33	**✓**	[147]
	Olive stone	-	PU	20	15.56	**✓**	-	**-**	[148]
	Peanut husks	-	UF	10; 8	9.9	**✕**	0.316	**✓**	[63]
	Peanut husks	pine chips	UF	10; 8	11.32	**✕**	0.35	**✓**	[63]
	Rice husks	-	UF	8	4.69	**✕**	0.04	**✕**	[105]
	Rice husks	Bamboo	UF	8	6.74	**✕**	0.07	**✕**	[105]
	Soybean husks	-	UF	10	11.02	**✕**	0.23	**✕**	[149]
	Soybean husks	ind. wood chips	UF	10	20.84	**✓**	0.40	**✓**	[149]
	Sugar beet pulp (CL)	-	UF	10; 7	6.29	**✕**	0.51	**✓**	[150]
	Sugar beet pulp (CL)	ind. wood chips	UF	10; 8	9.97	**✕**	0.51	**✓**	[150]
	Sugarcane bagasse	-	-	-	6 *	**✕**	0.01 *	**✕**	[151]
	Sugarcane bagasse	-	pMDI	3	16	**✓**	0.86	**✓**	[152]
	Sugarcane bagasse	-	pMDI	8	40 *	**✓**	1.8 *	**✓**	[151]
	Sugarcane bagasse (CL)	-	UF	10; 8	17 *	**✓**	0.42 *	**✓**	[107]
	Sugarcane bagasse (CL)	ind. wood chips	UF	10; 8	17.5 *	**✓**	0.45 *	**✓**	[107]
	Walnut husks	-	UF	11; 9	5.86	**✕**	0.24	**✓**	[153]
	Walnut husks	ind. wood chips	UF	11; 9	8.62	**✕**	0.34	**✓**	[153]
	Waste tea leaves	-	UF	8	37 *	**✓**	0.16 *	**✕**	[154]
	Waste tea leaves	ind. wood chips	UF	8	35 *	**✓**	0.22 *	**✕**	[154]

* derived from figure, CL (core layer), ind. (industrial), MUF (melamine urea-formaldehyde), MUPF (melamine urea phenol-formaldehyde), PF (phenol-formaldehyde), pMDI (polymeric diphenylmethane diisocyanate), PU (polyurethane), and SL (surface layer).
